# Supplementary figures and images for: The Clock:Cycle complex is a major transcriptional regulator of Drosophila photoreceptors that protects the eye from retinal degeneration and oxidative stress
Source: PLoS Genet. 2022 Jan 31;18(1):e1010021. doi: 10.1371/journal.pgen.1010021 (PMC8830735; doi:10.1371/journal.pgen.1010021)

**A**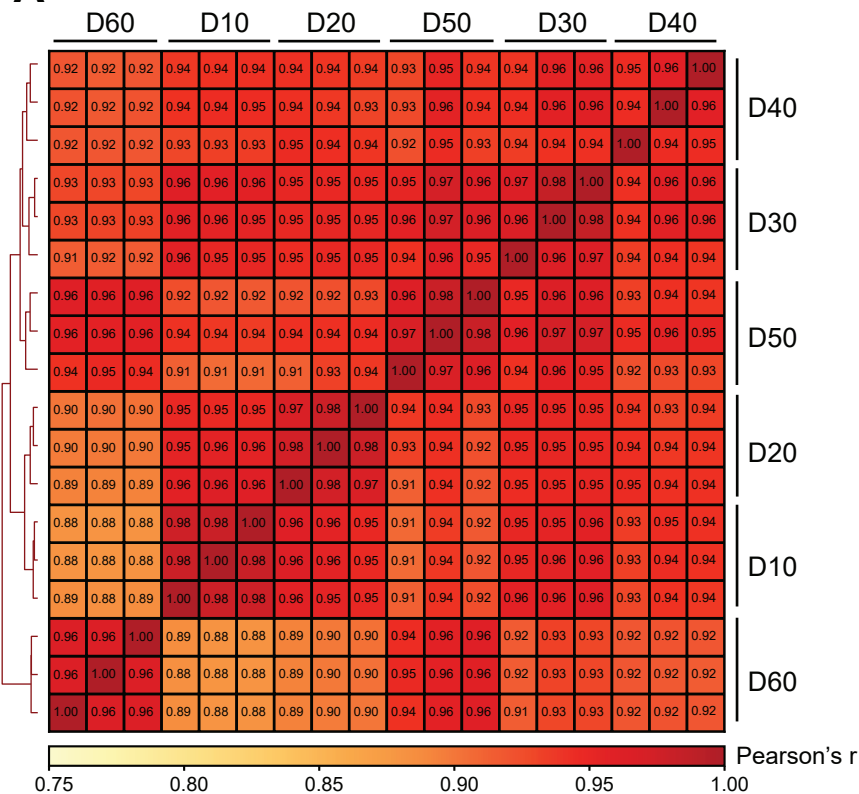

Supplement: S1 Fig — Pearson correlation heatmap of gene expression profiles from nuclear RNA-seq of aging samples (n = 3). Scores between 0 and 1 shown in each box correspond to Pearson’s r score. (PDF) [file pgen.1010021.s001.pdf]

**A****Age (days)**  
10 20 30 40 50 60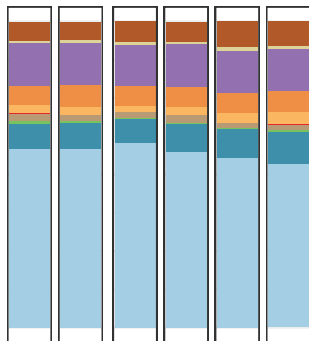**Feature**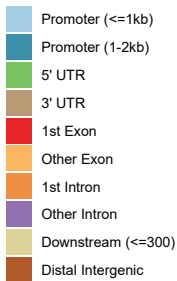**B****Clk/cyc binding motif**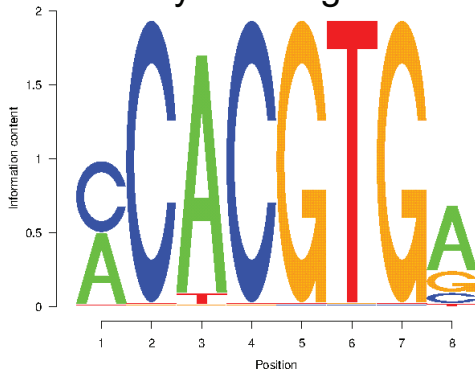

Supplement: S2 Fig — A. Bar plot showing the genomic distribution of accessible peaks identified in ATAC-seq data in Rh1>GFPKASH during aging. Promoter annotation was based on -/+ 2 kb around transcription start sites. B. DNA binding motif for Clk and Cyc derived from CIS-BP database. (PDF) [file pgen.1010021.s002.pdf]

**A**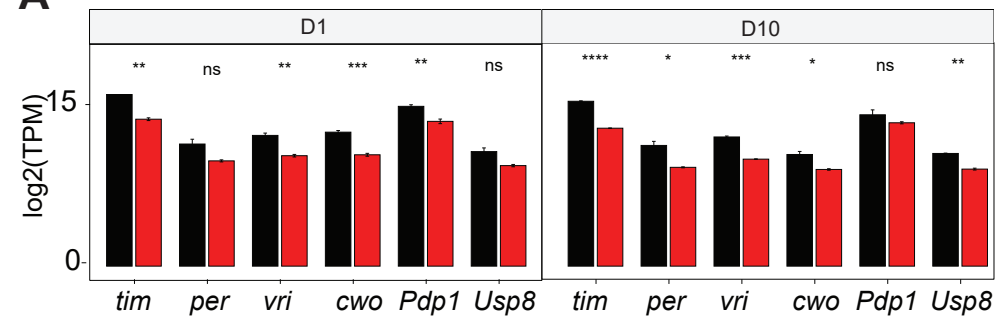**B**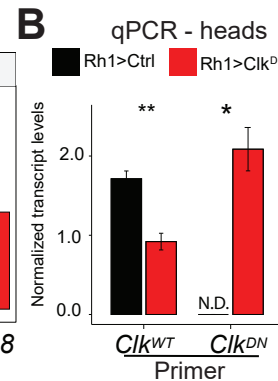**C**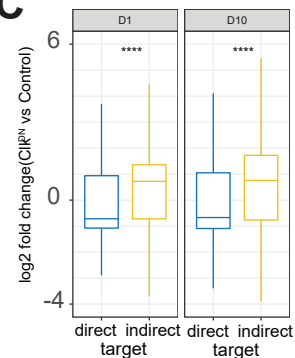**D**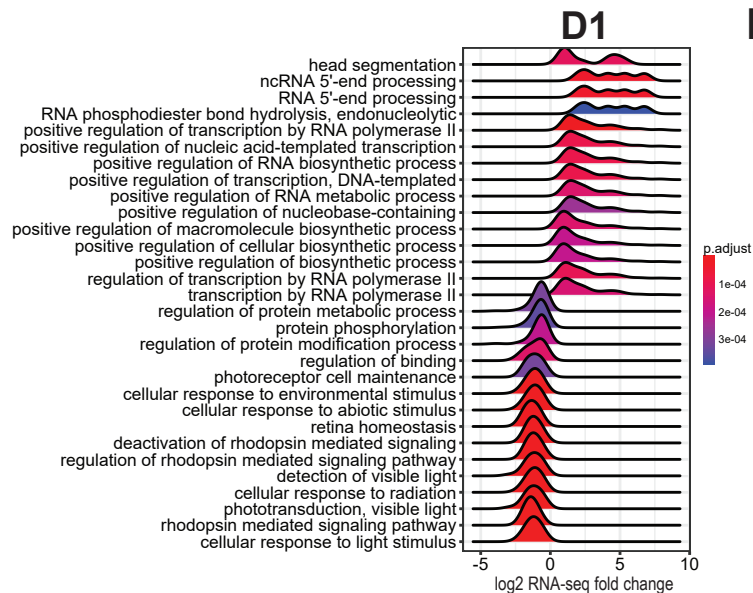**E**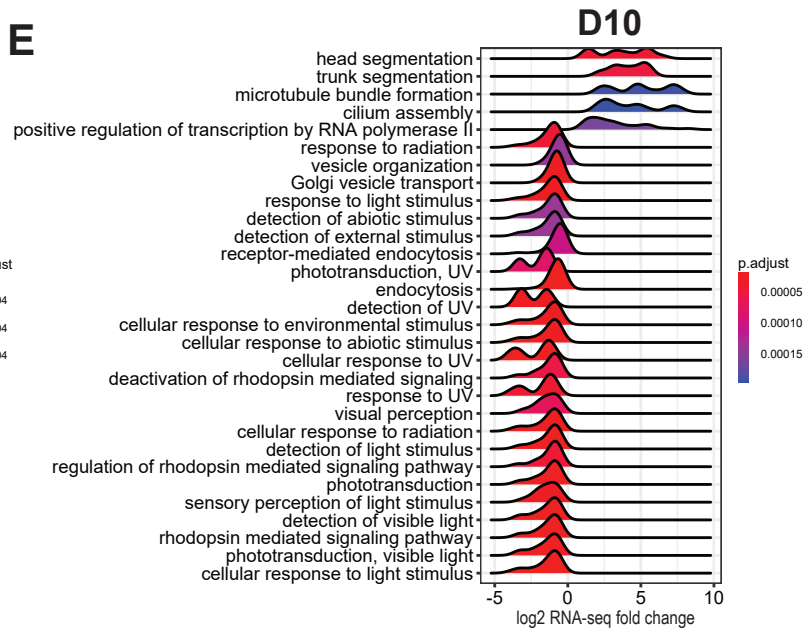**F**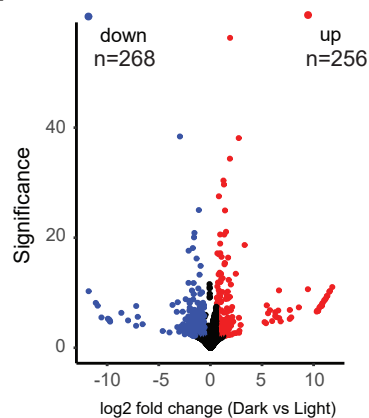**G**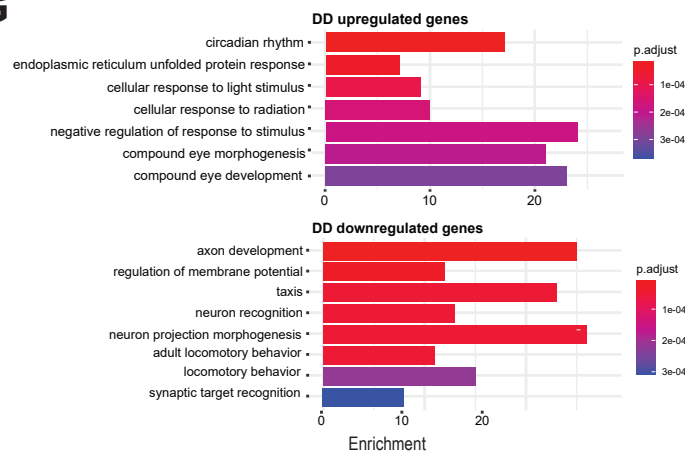

Supplement: S3 Fig — A. Bar plot showing log2-transformed transcript per million (TPM) values for core Clock genes comparing Rh1>ClkDN and Rh1>Ctrl at either D1 (left) or D10 (right) (mean +- SD, n = 3, P-value obtained using t-test). B. Bar plot showing ClkWT and ClkDN transcript levels measured in heads from male flies of the indicated genotype. Transcript levels are normalized to the geometric mean of housekeeping genes eIF-1α and RpL32 (mean ± SD; n = 3, P-value obtained using t-test). “ND” = not detected, “*” = p-value<0.05, “**” = p-value<0.005. C. Box plots showing the fold change values for genes identified as direct or indirect targets. Fold change is obtained using DESeq2. P-adjusted > 0.05, |FC|>1.5. P-value is obtained using Wilcoxon test. D,E. Complete ridge plot obtained from Gene Set Enrichment Analysis comparing differentially expressed genes in Rh1>ClkDN relative to Rh1>Ctrl at D1 (D) or D10 (E). F. Volcano plot showing differentially expressed genes from photoreceptors when flies are reared in free-running conditions (dark:dark) versus light:dark cycle. Significance is equivalent to -log10(adjusted p-value). Genes are colored based on changes in gene expression in D:D relative to L:D. Red is upregulated, and blue is downregulated. DEGs have p-adj < 0.05. G. Gene Ontology (GO) term analysis of genes that were significantly up-regulated (top) or down-regulated (bottom) in D:D relative to L:D. (PDF) [file pgen.1010021.s003.pdf]

**A**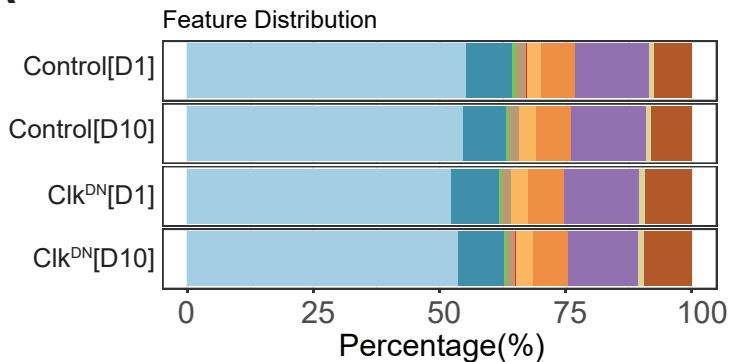**B**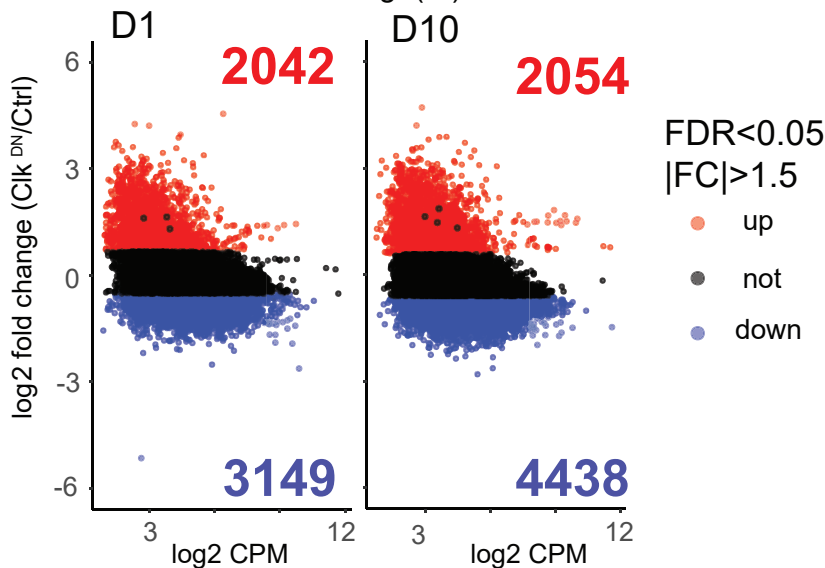

Supplement: S4 Fig — A. Bar plot showing the genomic distribution of accessible peaks identified in ATAC-seq data from the indicated genotypes and ages. Promoter annotation was based on -/+ 2 kb around transcription start sites. B. Volcano plots representing the differentially accessible peaks in Rh1>ClkDN relative to Rh1>Ctrl. Differentially accessible peaks are defined as having a False Discovery Rate (FDR) < 0.05, and absolute fold change (|FC|) > 1.5. (PDF) [file pgen.1010021.s004.pdf]
