# Supplementary material for: The Clock:Cycle complex is a major transcriptional regulator of Drosophila photoreceptors that protects the eye from retinal degeneration and oxidative stress
Source: PLoS Genet. 2022 Jan 31;18(1):e1010021. doi: 10.1371/journal.pgen.1010021 (PMC8830735; doi:10.1371/journal.pgen.1010021)
Supplement: S5 Fig — A. Heatmap showing RNA-seq fold change values for genes associated with phototransduction in flies expressing Rh1>ClkDN relative to Rh1>Ctrl at D1 or D10. Fold change values are represented inside each square, and color-labelled based on fold change score. B. Images corresponding to representative flies from the indicated genotypes used to assess retinal degeneration via optical neutralization. Only males are shown, but females also showed similar eye pigmentation. C. Representative images of eyes from flies expressing CycDN at the indicated age reared in light:dark (LD) conditions. D. Clk and cyc transcript ratios in aging samples. Kruskal-wallis test was used to determine differences amongst all groups, and pair-wise comparisons were obtained using standard t-test. No comparison was significant. (PDF) [file pgen.1010021.s005.pdf]

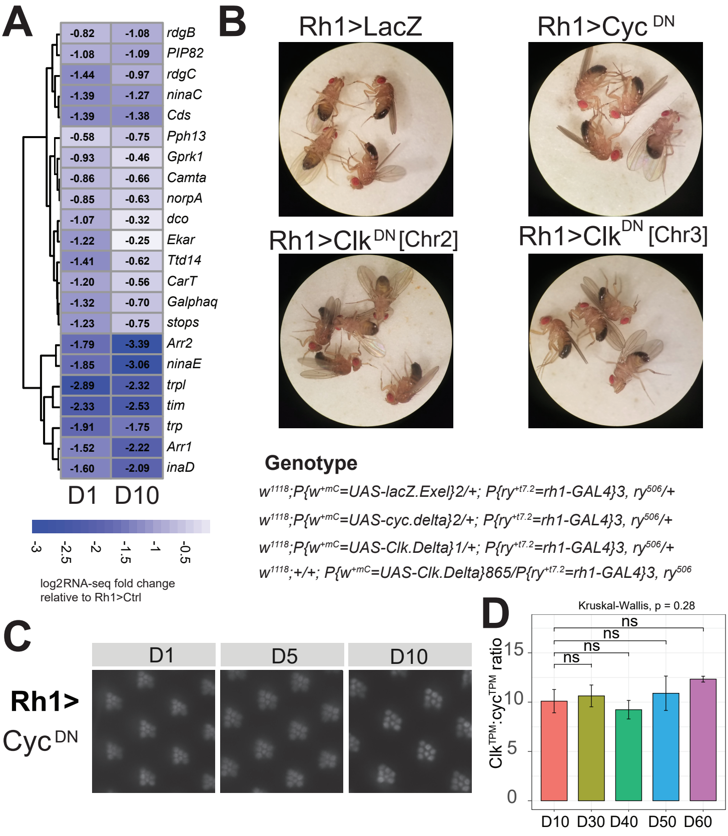
A
-0.82 -1.08 rdgB
-1.08 -1.09 PIP82
-1.44 -0.97 rdgC
-1.39 -1.27 ninaC
-1.39 -1.38 Cds
-0.58 -0.75 Pph13
-0.93 -0.46 Gprk1
-0.86 -0.66 Camta
-0.85 -0.63 norpA
-1.07 -0.32 dco
-1.22 -0.25 Ekar
-1.41 -0.62 Ttd14
-1.20 -0.56 CarT
-1.32 -0.70 Galphaq
-1.23 -0.75 stops
-1.79 -3.39 Arr2
-1.85 -3.06 ninaE
-2.89 -2.32 trpl
-2.33 -2.53 tim
-1.91 -1.75 trp
-1.52 -2.22 Arr1
-1.60 -2.09 inaD
D1 D10
-3 -2.5 -2 -1.5 -1 -0.5
log2RNA-seq fold change
relative to Rh1>Ctrl
B
Rh1>LacZ
Rh1>Cyc DN
Rh1>Clk DN [Chr2]
Rh1>Clk DN [Chr3]
Genotype
w1118;P{w+mC=UAS-lacZ.Exel}2/+; P{ry+t7.2=rh1-GAL4}3, ry506/+
w1118;P{w+mC=UAS-cyc.delta}2/+; P{ry+t7.2=rh1-GAL4}3, ry506/+
w1118;P{w+mC=UAS-Clk.Delta}1/+; P{ry+t7.2=rh1-GAL4}3, ry506/+
w1118;+/+; P{w+mC=UAS-Clk.Delta}865/P{ry+t7.2=rh1-GAL4}3, ry506
C
D1
D5
D10
Rh1>
Cyc DN
D
Kruskal-Wallis, p = 0.28
ns
ns
ns
ns
Clk TPM:cyc TPM ratio
15
10
5
0
D10 D30 D40 D50 D60
