# Supplementary material for: The Clock:Cycle complex is a major transcriptional regulator of Drosophila photoreceptors that protects the eye from retinal degeneration and oxidative stress
Source: PLoS Genet. 2022 Jan 31;18(1):e1010021. doi: 10.1371/journal.pgen.1010021 (PMC8830735; doi:10.1371/journal.pgen.1010021)
Supplement: Attachment — Submitted filename: Jauregui_ReviewerResponse_2021.pdf [file pgen.1010021.s011.pdf]

RESPONSE TO REVIEWERS: Manuscript ID: PGENETICS-D-21-01242

We would like to thank the editor and reviewers for their comments and suggestions. We have addressed each comment with our specific responses below (italics). We have made changes to the manuscript text as suggested by each Reviewer. We provide the page and line numbers for each of these changes in the text under the response to each comment below (red italics). Changes to the revised text are highlighted in yellow on the marked resubmission.

Except for minor formatting changes (*eg* CLK and CYC have been changed to Clk and Cyc in all figures), there are no major changes to Figures 1 – 4 from the original submission. Figure 5 has been revised to present the data more clearly, and we also now show the optic neutralization data from two independent Clk^{DN} lines in this figure. Supplemental Figure 5 has been revised to include photographs of flies used for optic neutralization studies, the Cyc^{DN} optic neutralization results and a bar plot showing the *Clk* vs *Cyc* transcript ratio. We have also added a new supplemental table that contains all the gene names/symbols and Flybase IDs corresponding to any genes or proteins referred to in this study.

Reviewer #1:

Comment#1: Perhaps the authors should discuss a little bit more the photoreceptors in Drosophila and why they decide to focus on the outer photoreceptors

We focused on the outer photoreceptors (R1 – R6 cells) because these are analogous to vertebrate rods, are the most abundant photoreceptor cell type in the fly retina, and are responsible for motion vision and phototaxis. In contrast, the inner photoreceptors R7 and R8 are responsible for color vision in flies. We have briefly expanded our description of the Drosophila eye and outer photoreceptors in the introduction section to address this point. See Introduction, page 4 lines 72-75.

Comment#2: In reading the manuscript I was totally confused by the nomenclature used by the authors with respect to the gene and protein. sometime the genes are in Italian with the first letter in capital sometime there are not the same is true for protein. They authors need to be consistent and they must use the appropriate nomenclature for Drosophila.

We apologize for the non-standard use of gene nomenclature/terminology in the original submission, and this point was also raised by Reviewer #3. We have now updated the entire manuscript (text and figures) to reflect standard nomenclature for gene/protein names as described in Flybase. Briefly, gene names and symbols are shown in italics (eg cycle; cyc) with corresponding proteins shown non-italicized with the first letter capitalized (eg Cycle; Cyc*). We refer to transcription factors identified using diffTF by their protein name. We have also added a new supplemental table (Table S3) with the Flybase IDs, gene names and symbols for all genes/proteins referred to in the text. See new Supplemental Table S3.*

Comment #3: The authors need to provide more details about the statistical analysis used to determine difference in the gene expression. The sample size of the sample

(n=3) does not really provide assurance about the rigor and reproducibility of the data. Therefore it is important that the statistical methodology used to determine differences is well explained in the manuscript.

We now provide additional details about the statistical analysis used for analyzing differential gene expression. For each biological replicate for high-throughput experiments (RNA-seq, ATAC-seq), we collected 400 age-matched male flies and performed one NIE reaction per replicate (n = 3 biological experiments). Three biological replicates is acceptable for differential gene expression analysis if there is sufficient sequencing depth (PMID: 24020486), and we routinely use this experimental design for RNA-seq experiments (PMID: 30029619, 30003673, 34022041, 29162050). We agree that additional biological replicates are helpful for these analyses, but this can be difficult to achieve in practice when there are multiple conditions evaluated (eg ages) and each sample represents a pool of hundreds of individual flies. We have now modified Figure 1, results, and methods sections to full describe the number of flies used per biological replicate for RNA-seq and ATAC-seq experiments. See Figure 1, Results line 107-108, and Methods line 559-560.

Comment #4: The authors should expand a little the section in which they are comparing the data to the mouse. For example a recent paper in the mouse has shown that removal of the Bmal1 gene lead to change in the spectral identity of the cone photoreceptors by modulating the expression of a gene coding for an hormone (Sawant OB, et al. 2017 The circadian clock gene Bmal1 controls thyroid hormone mediated spectral identity and cone photoreceptor function. Cell Rep 21:692–70).

We thank the reviewer for bringing this paper to our attention and we now cite this study in the discussion section, and more broadly describe the role of the circadian clock in regulating vertebrate retinal homeostasis. See Discussion section, line 441-447

Reviewer #2:

Comment #1: Why should Clock activity change as flies age? Is this because the circadian rhythm changes? Or is it because Clock is activated by damage/injury/stress independent of the circadian rhythm? Simple measurements of the circadian rhythm of the aging flies might provide useful clues.

This is a very interesting question, and we now expand our discussion of the potential mechanisms underlying the increase in Clock activity in aging photoreceptors in the Discussion. Briefly, a study that profiled steady state RNA from fly heads at D5 and D55 (PMID: 28221375) showed that Clock transcriptional targets, such as tim and per, show an overall increase in the amplitude of transcription, in addition to a slight shift in the phase, moving earlier as flies aged. This same study also observed an overall decrease in PER protein levels in old flies, and this decrease in PER levels was consistent with immunostaining results from another paper (PMID: 22217096). We speculate that there is an age-associated decrease in PER levels in the eye of old flies, although we have not tested this hypothesis in the current study. We are actively pursuing this question in our group; please see also response to Comment #2. See Discussion, lines 505-510, 513-517.

Comment #2. In the discussion, the authors speculate that aging could change the phase of Clock activity, or the amplitude. I believe that this could be tested experimentally. For example, the authors could assess Clock target gene expression at multiple time points throughout the day. They could compare the phase and amplitude of Clock target genes in young versus old flies.

We absolutely agree that characterizing age-associated changes to the circadian clock in photoreceptors (phase/amplitude) is one of the most interesting questions arising from our study, but we believe that to answer this in a rigorous manner will require extensive experiments that are well beyond the scope of this manuscript. We are planning to conduct our own experiments to examine this question more carefully because the published studies that we refer to in our response to comment #1 used steady-state RNA from whole heads (and the circadian clock genes are highly regulated at post-transcriptional levels), only had 2 biological replicates, and used w^{1118} flies, which are white-eyed flies that are highly sensitive to light-mediated damage (PMID: 28221375). We are in the process of beginning a series of experiments to profile the photoreceptor transcriptome (as described in the current manuscript) at different time points in the day (every 4 hours) in 10-day and 50-day old flies. Since our method profiles nuclear RNA, we believe that this approach will provide a better proxy to identify transcriptional targets of Clk:Cyc that are specific to photoreceptors, undiluted by other cell types in the head. However, we believe that these experiments are beyond the scope of the current work. We thank the reviewer for this suggestion and we hope to present the results of these follow-up studies soon. See also response to comment #1 and Discussion, line 505-510, 513-517.

Comment #3: The authors rely on Clock DN expression to assess Clock's role in neuroprotection. It would be nice to independently validate phenotypes of Clk DN through other means (just to rule out off-target or genetic background effects). For example, does Clock RNAi also cause light-dependent retinal degeneration?

We initially screened retinal degeneration (RD) in flies that overexpressed Clk^{DN} in photoreceptors, using two publicly available lines from BDSC consisting of UAS-Clk^{DN} insertions on either Chr2 or Chr3 (#36318 and #36319), originally described in PMID: 15084278. Both independent Clk^{DN} lines resulted in progressive light-dependent RD, suggesting that the phenotype results from the overexpression of the dominant negative mutant, rather than off-target or genetic background effects. However, we agree that examining other flies in which Clk:Cyc activity is disrupted would be informative, and to that end we obtained UAS-Cyc^{DN} from Dr. Daniel Cavanaugh at LoyolaU. When we performed optic neutralization in Rh1>Cyc^{DN} flies, we did not observe any RD at D5 or D10. We confirmed the identity of the Clk^{DN} and Cyc^{DN} by PCR, and all these lines are correct. One potential explanation for the difference we observe is that Clk nuclear transcripts are 10-times higher that cyc (TPM analysis). In addition, mass spectrometry analysis from fly heads showed that overall, Clk peptides are 5-times more abundant than Cyc peptides during the day (PMID: 33087840). Therefore, we speculate that the absence of RD phenotype upon overexpression of Cyc^{DN} could result from Clk^{WT} vs Cyc^{WT} protein levels i.e. insufficient levels of Cyc^{DN} to bind and sequester Clk^{WT}. However, an alternative interpretation is that Clk and/or Cyc have independent functions and perhaps TF binding partners in photoreceptors. As a first step in distinguishing

between these possibilities, we would like to identify genome-wide binding sites for Clk and Cyc specifically in photoreceptors – and we are currently pursuing approaches to identify these sites, which should provide an indication of whether Clk and Cyc bind any regions independently. We describe the UAS-Cyc^{DN} data in the supplemental material for Figure 5, and have updated the Results section to reflect these findings. See Figure 5, Results, line 369-388 and Supp Figure S5.

Comment #4. Is there an effect of over expressing wild type Clock in photoreceptors? Would such conditions enhance neuroprotection? Alternatively, would it cause retinal degeneration because it disrupts the circadian clock?

Unfortunately given the time constraints and current shipping difficulties, we were not able to obtain the available UAS-Clk-3xHA or UAS-Cyc-3xHA flies from the Zurich ORF collection in time to cross these and age out to D50 – when wild-type flies start to exhibit retinal degeneration. A 2019 study showed that over expression of several Clock genes, including Cyc^{WT} using pan-neuronal driver Elav-Gal4 led to an increase in longevity (PMID: 30415070), suggesting that enhancing the positive transcriptional loop in the Circadian clock might have a neuroprotective effect in flies. Supporting this, and as described in the Discussion section, tim^{01} and per^{01} also have increased longevity (PMID: 32317636). See Discussion, line 531 – 534.

Comment #5. The Methods section doesn't describe in sufficient detail how the authors performed the retinal degeneration assay. Were the flies in the w+ or w- background? Were there similar amounts of eye pigments in control versus Clock DN flies? Was retinal degeneration assessed through immunostaining?

We have now expanded our methods section to fully describe how we performed optical neutralization of the cornea to assay retinal degeneration. Briefly, bright-field microscopy is used to directly assess rhabdomere integrity in the eye of live flies without the need to perform antibody-based staining. Fly heads are mounted on a slide with a drop of nail polish and illuminated, enabling us to direct visualize the rhabdomere tips. One of the caveats of this technique is that flies must have pigmented eyes (orange/red), which is the case for the flies used in this study because all flies have mini-white [w^{+mC}] transgenes. We show photographs of representative flies and genotypes in Figure Supplemental 5. We note that in our lab, w^{1118} flies start showing signs of retinal degeneration at fairly young ages (~D15), as assessed by phalloidin staining, so we avoided using this genetic background in the current study. See Results, line 362-364, Methods, line 592-596, and Figure Supplemental 5.

Comment #6. Figure 5B has representative images of eyes in control and Clock DN flies. The legend does not say whether this is an immunohistochemical image (or not), and what antibody was used.

Retinal degeneration was assessed using optical neutralization by brightfield microscopy, with no immunostaining required. We have revised the figure legend to reflect the technique used more clearly. See also response to comment #5. See Figure Legend for Figure 5, line 1000-1001.

Reviewer #3:

Comment #1: They have found that some genes are up-regulated and other are down-regulated and these are rather expected results. In this part of the study I did not learn about mechanisms of aging.

The overall aim of the integrated RNA-seq and ATAC-seq approach in aging photoreceptors was to identify changes in transcription factor binding activity that could drive the changes that we observe in the aging transcriptome. We previously profiled the aging photoreceptor transcriptome (PMID: 29162050) and we described the types of gene expression changes observed in detail in this previous study, showing that they correlate with a decline in visual behavior. Although we broadly describe the RNA-seq changes in gene expression in Figure 1, this description is intended to simply provide an overview of the data and an indication of its quality for the diffTF analysis.

Comment #2: In the second part of the paper the Authors analyzed the role of the circadian clock on gene expression in R1-R6 in 1 day and 10 days old flies. In this part of the study it is unclear if the Authors tried to learn about the expression of clk and cyc levels or the effect of the circadian clock disruption in R1-R6 photoreceptors. If the last is true, studying the clock effects on gene expression only at one ZT time points is not enough.

We absolutely agree that examining multiple ZT time points would be interesting; however, this was beyond the scope of the current study (see also responses to Reviewer #2 comments #1 and #2). In our study, we were interested in understanding the consequences of disrupting the Clk:Cyc complex. We reasoned that performing these studies at ZT9 would provide the best pair-wise comparison between Clk^{DN} and control because ZT9 is a well-stablished timepoint to study Clock-dependent transcription. Further, expression of the Clk^{DN} mutant in Clock or antennal neurons consistently abolishes rhythmic transcription and behaviors (PMID: 15084278). Thus, analyzing differences in transcript/chromatin level at ZT9 provides an indication of how Clk^{DN} affects Clock-dependent transcription in the cell type of interest for our study, photoreceptors.

Comment #3: The Authors compare results from the first part of the study with the second part but they examined flies in different age and collected them at the different time point. Moreover, it is well known that many genes are clock-controlled and the disruption of the clock leads to changes not only at the level of transcription but also at posttranscriptional processes, translation, posttranslational processes and on many other processes in the cell.

We agree that given the differences in sample collection for the aging (ZT6) and CLK^{DN} (ZT9) experiments, it is unacceptable to directly compare RNA-seq data using normalized CPMs in order to draw conclusions about what happens in aging (where our data shows increased Clk:Cyc TF activity), versus CLK^{DN} (which shows decreased Clk:Cyc TF activity). Therefore, we chose to only speculate about these similarities/comparisons in the Discussion section. Nonetheless, given that our starting material for the high-throughput experiments is nuclear RNA (because of the NIE procedure), our data allows to obtain a snapshot of the effect of disruption the circadian clock on the nascent transcriptome. Importantly, a previous study of the nascent transcriptome in fly heads revealed that not all rhythmic genes at a nascent RNA level

are rhythmic when evaluated using mRNA-seq (PMID: 23297234). We believe that our RNA-seq analysis provides a closer measure of transcription because we profile nuclear transcripts, thus avoiding some of the issues you mention regarding post-transcriptional regulation.

Comment #4: In case of photoreceptors, it is known that the phototransduction is controlled by the peripheral clock in photoreceptors and by light and degeneration of the retina results not only from disruption of the clock but also from DNA brakes induced by UV and white light.

While many stressors (such as increased DNA damage, reactive oxygen species) can lead to retinal/neurodegeneration, our data suggest that misregulation of phototransduction gene expression is the most likely explanation for the retinal degeneration induced by photoreceptor-specific expression of Clk^{DN}. The strongest evidence for this comes from our observation that Clk^{DN}-dependent retinal degeneration can be suppressed by raising flies in constant darkness, which mimics the phenotype of many phototransduction mutants. Secondly, our data argue against a role for ROS because glutathione redox ratios still showed signs of oxidative stress even in the dark where there was no retinal degeneration. However, we are aware that many environmental stressors including blue light exposure contribute to retinal degeneration in flies, and examining the connections between the circadian clock and stress response is an interesting future area of investigation for our group.

Comment #5: There are also problems in terminology and it is unclear when the Authors write about the clock, genes clock and cycle and when about proteins CLK and CYC.

We apologize for the confusing use of gene nomenclature/terminology, and this point was also raised by Reviewer #1. We have now updated the entire manuscript to reflect a more accurate use of nomenclature for gene/protein names. We also provide new supplemental Table S3 with a list of all genes/proteins and their corresponding Flybase IDs. See supplemental Table S3.

Comment #6: For the second part of the paper the Authors used only young flies, 1 and 10 days old, so the results do not show aging in the photoreceptors.

We agree with the reviewer that the experiments in Clk^{DN} flies, which show decreased Clk:Cyc activity at least based on transcript levels for the canonical Clock targets tim, per and vri, do not represent normal aging. In fact, based on the bioinformatic analysis, our data shows that in normal aging, photoreceptors have increased rather than decreased Clk:Cyc activity. Thus, we propose that Clk:Cyc activity is normally required to prevent retinal-degeneration during normal aging, by promoting gene expression programs that protect retinal cells from different sources of stress, potentially including Rh1 misfolding and oxidative stress.

Comment #7: Everywhere in the text it is not clear what does it mean "loss of Clock activity", it is gene, protein or the circadian clock.

We originally described expression of Clk^{DN} as "loss of Clock activity", since Clock transcriptional targets had decreased transcript levels in Clk^{DN} relative to control flies. However, to provide a more accurate description of the molecular consequences of expression of Clk^{DN}, we have now modified the manuscript and replaced the use of

"loss of Clock activity" to "expression of Clk^{DN}" throughout the text. See text changes highlighted in yellow in Introduction, Results and Discussion.

Comment #8: P. 4 The Authors "identified 61 TFs with substantial changes in activity during aging", however, they have not analysed proteins but genes.

The diffTF bioinformatic analysis used to identify changes in TF activity estimates changes in "TF activity" by measuring genome-wide changes in chromatin accessibility around transcription factor binding sites. This estimation is used as a proxy for "TF activity", or more accurately, "TF binding/motif activity". We now expand this definition in the text. Thus, the TFs identified represent proteins rather than genes – because diffTF identifies changes in the use of their corresponding binding sites (or motifs) within the genome. See Results, lines 150, 152-155.

Comment #9: P. 4 According to the Authors: "Our data identify a novel neuroprotective role for the circadian clock in the Drosophila eye, and suggest that this role may become increasingly critical in advanced age to prevent retinal degeneration." I disagree with this statement since degeneration of the retina may also depend on desynchronization of rhythms in different cells in the retina. Moreover, the Authors did not show that overexpression of Clk and cyc in 10 days old flies protects the photoreceptors against degeneration.

We agree that desynchronization of rhythms in different cells in the retina could contribute to retinal degeneration. and this is very well established in vertebrate systems, where it known that most cells contain circadian clocks. However, in Drosophila, photoreceptor cells are amongst the tissues that contain a working circadian clock (PMID: 24405673). In addition, we express Clk^{DN} specifically in outer photoreceptor neurons (R1 – R6 cells) using Rh1-Gal4, and this photoreceptor-restricted expression leads to retinal degeneration. Thus, our data demonstrates that photoreceptor-specific disruption of Clock activity is sufficient to produce light-dependent retinal degeneration. We were unable to obtain the flies necessary to overexpress wild-type Clk (see Reviewer #2 comment #4) in sufficient time to cross and age these out to D50 – D60 when wild-type flies begin to show retinal degeneration. However, this is an interesting future experiment and we thank the reviewer for this suggestion.

Comment #10: The following paragraph title: "Clock activity promotes changes in TF activity and maintains global levels of accessibility in photoreceptors" is unclear. What does it mean "activity" in both cases?

To improve readability of the manuscript and contain more accurate description of the molecular system used in the manuscript to study disruption of Clock activity, we have now updated to manuscript and use "Expression of Clk^{DN}" instead of "loss of Clock activity". However, we note that expression of Clk^{DN} in Clock or antennal neurons disrupts circadian transcription and rhythmic behaviors (PMID: 15084278). See text changes highlighted in yellow in Introduction, Results and Discussion.

Comment #11: P. 13, para 2, l. 2 What does it mean "chromatin accessibility around gene bodies"?

We mistakenly used "around" instead of "throughout", or "over" gene bodies. In this case, we refer to an open chromatin state over gene bodies, which is commonly seen for actively, and highly expressed genes. We have revised this sentence to make this point clearer. See Results, lines 327.

Comment #12: P. 16 Discussion l. 3. The eye is not required for circadian behaviour in flies but the retina photoreceptors contribute to the circadian behavior.

In Drosophila, retinal photoreceptors are not required to maintain circadian behavior, as glass mutant (gl^{60j}) flies, which lack compound eyes, can be entrained to the light:dark cycle (PMID: 11343659). However, we agree that retinal photoreceptors contribute to circadian behavior. We have updated the text to more accurately describe this point. See Discussion, line 434-435

Comment #13: P. 16 Discussion l. 5. It is possible that the effect of the circadian clock disruption on retinal degeneration is not direct.

We agree that there could be circadian-clock independent roles of Clock and/or Cycle in retinal degeneration, and this possibility is supported by lack of retinal degeneration caused by Cyc^{DN}. We describe this possibility in the results and discussion. See Discussion, line 463-466.

Comment #14: P.16 Discussion. In case of BMAL1 many functions have been found which are not clock-dependent.

We agree that Clock-independent functions of BMAL1 could underly the gene expression and retinal degeneration phenotypes observed in this study, and we describe this possibility with some supporting literature in the results and discussion. See Discussion, line 463-466.

Comment #15: Fig. 5 B Representative images of eyes…. Unfortunately, degenerative changes are not visible in this figure.

We have now modified Figure 5B and added white arrows to indicate the position of the missing rhabdomeres. Quantification of these data are also provided in Figure 5C. See revised Figure 5.
