# Supplementary material for: The Clock:Cycle complex is a major transcriptional regulator of Drosophila photoreceptors that protects the eye from retinal degeneration and oxidative stress
Source: PLoS Genet. 2022 Jan 31;18(1):e1010021. doi: 10.1371/journal.pgen.1010021 (PMC8830735; doi:10.1371/journal.pgen.1010021)
Supplement: Attachment — Submitted filename: ReviewerResponse_2_Jauregui_2022.pdf [file pgen.1010021.s012.pdf]

RESPONSE TO REVIEWERS: Manuscript ID: PGENETICS-D-21-01242R1

We would like to thank the editor and reviewers for their comments and suggestions, and we are glad that our changes to the previous version fully addressed the original comments raised by the first and second reviewers.

In this version, Reviewer #3 raised several questions and we have addressed each of these comments with our specific responses below (italics). We have made one change to the Discussion section as suggested by Reviewer #3, minor comment #3. We provide the page and line numbers for this change in the text under the response to each comment below (red italics). Changes to the revised text are highlighted in yellow on the marked resubmission. There are no other changes to the figures or supplemental material in this version.

In addition, we have added the link in the Methods sections to a protocols.io protocol detailing step-by-step protocols for nuclei immuno-enrichment, RNA-seq, and Omni-ATAC, as performed in the present manuscript. We also removed the reviewer's token information to access the GEO databases since they are now released. We also corrected any minor errors in the text in this version (highlighted in yellow).

Major points

1) The manuscript has been improved, however, because of the experimental procedure it still does not show which processes in R1-R6 photoreceptors depend on Clk as a component of the molecular mechanism of the circadian clock and which ones on clock independent Clk function.

We agree that this study does not identify which processes depend on Clk as a component of the circadian clock versus potentially independent function, but as we previously discussed in our response to Reviewer #2 in the previous resubmission (reviewer #2, comment #3):

"However, an alternative interpretation is that Clk and/or Cyc have independent functions and perhaps TF binding partners in photoreceptors. As a first step in distinguishing between these possibilities, we would like to identify genome-wide binding sites for Clk and Cyc specifically in photoreceptors – and we are currently pursuing approaches to identify these sites, which should provide an indication of whether Clk and Cyc bind any regions independently."

Thus, we believe that in order to be able to determine which processes in photoreceptors are Clock-dependent vs -independent, we would need to perform additional series of experiments, which would require substantially more time and are beyond the scope of the present manuscript. We agree that this is an extremely important question, and we thank the reviewer for this suggestion.

2) There is also lack of experimental data explaining why expression of clk and cyc increases in old flies.

We agree that we do not yet understand the mechanism that underlies the increased activity of Clk:Cyc in old photoreceptors, and that this remains an important question for future studies. As we mentioned in our previous response to reviewer #2, comment #1 and #2: "This is a very interesting question, and we now expand our discussion of the potential mechanisms underlying the increase in Clock activity in aging photoreceptors in the Discussion. Briefly, a study that profiled steady state RNA from fly heads at D5 and D55 (PMID: 28221375) showed that Clock transcriptional targets, such as tim and per, show an overall increase in the amplitude of

transcription, in addition to a slight shift in the phase, moving earlier as flies aged. This same study also observed an overall decrease in PER protein levels in old flies, and this decrease in PER levels was consistent with immunostaining results from another paper (PMID: 22217096). We speculate that there is an age-associated decrease in PER levels in the eye of old flies, although we have not tested this hypothesis in the current study" and "We absolutely agree that characterizing age-associated changes to the circadian clock in photoreceptors (phase/amplitude) is one of the most interesting questions arising from our study, but we believe that to answer this in a rigorous manner will require extensive experiments that are well beyond the scope of this manuscript. We are planning to conduct our own experiments to examine this question more carefully because the published studies that we refer to in our response to comment #1 used steady-state RNA from whole heads (and the circadian clock genes are highly regulated at post-transcriptional levels), only had 2 biological replicates, and used w^{1118} flies, which are white-eyed flies that are highly sensitive to light-mediated damage (PMID: 28221375). We are in the process of beginning a series of experiments to profile the photoreceptor transcriptome (as described in the current manuscript) at different time points in the day (every 4 hours) in 10-day and 50-day old flies. Since our method profiles nuclear RNA, we believe that this approach will provide a better proxy to identify transcriptional targets of Clk:Cyc that are specific to photoreceptors, undiluted by other cell types in the head".

Although these mechanistic studies are beyond the scope of the current manuscript, they are indeed an important area of investigation and we thank the reviewer for this suggestion. If, as suggested by published data from other labs, protein levels for Tim and Per decrease with age specifically in photoreceptors – one key question is what drives this decrease?

3) I also do not understand why in adult flies, genes involved in the eye development (sevenless - expressed in R7, eyeless) show an increase in the expression with aging.

We apologize if the reviewer was confused about this point – but there is no data presented in the manuscript that shows an increase in expression of genes such as sevenless during aging. We did identify the DNA motif associated with TF eyeless (ey) as showing increased TF activity with age (diffTF analysis), and individual inspection of our TPM-normalized RNA-seq data for eyeless showed levels within 15-25 TPM demonstrating that eyeless is expressed through-out aging, albeit at relatively low levels (see below). We note that in our lab, we routinely use a cutoff of 5 TPMs for genes to be discarded as lowly to not expressed. We also inspected FlyAtlas2 (https://motif.mvls.gla.ac.uk/FlyAtlas2?search=gene&gene=ey&idtype=symbol#mobileTargetG) which showed that eyeless is enriched in the adult head, brain and eye (see below), further corroborating adult expression of eyeless. We note that for diffTF analysis, we used the entire database of TF motifs, since TF are normally lowly expressed and, are often under-detected in high-throughput analysis, such as RNA-seq and proteomics experiments. This happens to be one of the motivations for developing the diffTF software (2nd paragraph of original diffTF paper PMID: 31801079) since using changes in TF transcript/protein levels as proxy for changes in TF activity can be misleading. See figures below for reviewer use only describing expression of ey.

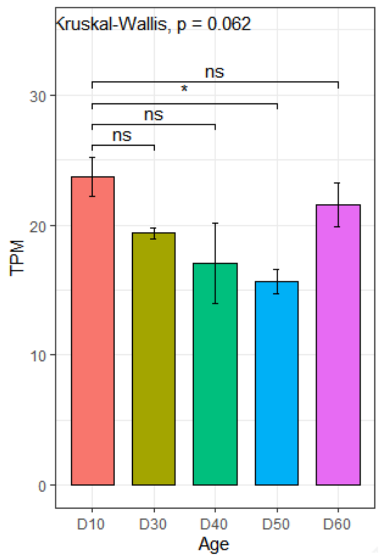

Symbol	Name	Annotation Symbol	FlyBase ID
ey	eyeless	CG1464	FBgn0005558

Gene FPKMs and Enrichments ☐ SDs ☐ Whol

	Adult Male		Adult Female	
Tissue	FPKM	Enrichment	FPKM	Enrichment
Head	6.5	3.3	6.0	3.0
Eye	9.9	5.0	7.4	3.7
Brain / CNS	12	6.1	11	5.5
Thoracicoabdominal ganglion	2.8	1.4	2.8	1.4

<u>Minor Comments:</u>

1) l. 273 Genes regulated by the Clk/Cyc heterodimer are called clock-controlled genes (ccg) but not clock-bound genes.

We agree with the reviewer that genes regulated by the Clk/Cyc heterodimer should be defined as CCG. However, binding of a TF in the promoter of a given gene does not necessarily reflect a function in gene regulation without integration of ChIP-seq/qPCR data with corresponding transcriptomic data. Thus, for our manuscript, we first define the Clock-bound gene category as genes expressed in photoreceptors (based on RNA-seq) that are bound by Clk and Cyc based on published ChIP-seq data obtained in fly heads (PMID: 24332542). Then, we perform an overlap of these "Clock-bound genes" with genes that are differentially expressed upon expression of Clk^{DN}, and refer to them as genes regulated by Clock in photoreceptors. These may be called as photoreceptor-specific clock-controlled genes, however, we found that definition to be cumbersome. We note that, to our knowledge, our study is the first study establishing which genes from the photoreceptor transcriptome respond to disruption of the Clk/Cyc complex. Nonetheless, this does not mean that these genes are not expressed or regulated by Clk/Cyc in other cell types, and we do not have data addressing that point in this study.

2) l. 415 There is several processes to protect cells against oxidative stress and the Authors should measure ROS level to examine oxidative stress but not one of those processes.

We agree that approaches that measure levels of specific types of ROS could be an alternative to measuring oxidized/reduced glutathione pools in this study. However, measuring the intracellular ratio of oxidized/reduced glutathione has been shown to work well as a proxy for relative levels of oxidative stress in several biological systems (PMID: 20700719, 23205122, 24809893). In addition, because the GSH:GSSG ratio did not correlate with the observed light-dependent retinal degeneration, we did not further pursuit the identification of different ROS upon expression of Clk^{DN}. However, we agree that identifying the specific ROS that increase upon expression of Clk^{DN} would be an interesting question, and we are developing some

approaches to examine other ROS species using immunostaining (eg H_2O_2, lipid peroxidation, live redox sensors). In our hands, we have found the GSH:GSSG ratios to be the most robust assay; we note that many of the live imaging redox sensors (eg roGFP-Grx) are also designed to measure changes in glutathione redox status as a proxy for oxidative stress.

l. 425 The Authors write: “We propose that Clock activity is protective in the retina because it promotes expression 20 of genes that contribute to proper recycling of Rh1 upon light-exposure.” I my opinion there are no data in the manuscript supporting this statement.

We present this statement as a potential hypothesis (using the language “propose”) rather than a conclusion based on the findings from this study – in particular our observations from RNA-seq that these 20 genes are down-regulated upon photoreceptor-expression of Clk^{DN} (Figure 4G-H, 6A). This hypothesis is further supported by the following data:

(1) these genes encode proteins that are necessary for Rh1 recycling from published literature
(2) RNA-seq data shows decreased transcript levels of phototransduction genes and increased transcript levels of genes associated with unfolded protein response upon expression of Clk^{DN}
(3) retinal degeneration observed upon expression of Clk^{DN} is dependent on light exposure

Based on this, we believe it is appropriate to propose this as a potential mechanism. We were careful not to present this as a conclusion in the study, but we believe that it is appropriate to discuss potential mechanisms in the Discussion section of the manuscript based on the data in this study.

3) l. 438 I disagree with the Authors’ statement that “the circadian clock plays a role in retinal homeostasis independent from maintenance of rhythmic behaviors”.
The rhythms in the retina are correlated with rhythms in behaviour. The difference between retinal and behavioural rhythms is that circadian rhythms in photoreceptors can be regulated by *the pacemaker, peripheral clocks inside photoreceptors or by both*

We apologize if this sentence was confusing or misleading. By stating that “the circadian clock plays a role in retinal homeostasis independent from maintenance of rhythmic behaviors”, we refer to the fact that we observed several molecular changes associated with transcription, in addition to light-dependent and light-independent phenotypes upon expression of Clk^{DN} specifically in photoreceptor neurons (using Rh1-Gal4). However, we understand that this statement could be misleading since we did not measure rhythmic behaviors in Rh1>Clk^{DN} flies, nor did we monitor if Rh1>Clk^{DN} disrupted the molecular clock in pacemaker neurons. Thus, we have now removed this statement from discussion and updated the first paragraph of the Discussion section, as shown below:

“Since many genes are rhythmically expressed in the retina including most of the phototransduction machinery (50,64,65), these observations suggest that the circadian clock plays a role in homeostatic regulation of the Drosophila retina.”

See Discussion section, page 21, lines 453-455.
