# Supplementary material for: The Clock:Cycle complex is a major transcriptional regulator of Drosophila photoreceptors that protects the eye from retinal degeneration and oxidative stress
Source: PLoS Genet. 2022 Jan 31;18(1):e1010021. doi: 10.1371/journal.pgen.1010021 (PMC8830735; doi:10.1371/journal.pgen.1010021)
Supplement: Attachment — Submitted filename: Jauregui_ResponseToReviewers_R3.docx [file pgen.1010021.s013.docx]

**RESPONSE TO REVIEWERS: Manuscript ID: PGENETICS-D-21-01242R2**

We would like to thank the reviewer for their comments and suggestions, and we are glad that our changes to the previous version addressed their concerns.

**Reviewer #3:** I accept the Authors' answers to my comments. The Authors's plan for the future studies sounds good, however, in my opinion their current results do not explain aging of the clock in the retina photoreceptors R1-R6 and how its aging affects aging of photoreceptors and their degeneration. The Authors report some changes and I can accept it as their first approach.

*We thank the reviewer and we are very interested in identifying the mechanism(s) underlying these changes in the circadian clock in aging photoreceptors.*

Minor comment #1: In addition the Authors should correct the first sentence in the Authors' Summary and instead "photoreceptor neurons" (l.43) write the retina outer photoreceptors.

*We have modified the summary as suggested. See Author Summary, page 2, line 42-43.*
